# Effects of inhaled anesthetics sevoflurane and isoflurane on lung morphofunction and biological markers in experimental pulmonary and extrapulmonary acute respiratory distress syndrome

**DOI:** 10.1186/2197-425X-3-S1-A571

**Published:** 2015-10-01

**Authors:** CS Samary, MN Araujo, CL Santos, FF Cruz, VM Cavalcanti, LB Heil, FC Fernandes, NR Vilela, PL Silva, PR Rocco

**Affiliations:** Federal University of Rio de Janeiro, Carlos Chagas Filho Biophysics Institute, Rio de Janeiro, Brazil; Federal University of Rio de Janeiro, Rio de Janeiro, Brazil

## Introduction

Experimental and clinical studies have shown that volatile anesthetics modulate the inflammatory process, which may be beneficial in the context of acute respiratory distress syndrome (ARDS). Furthermore, these agents may also protect type II epithelial cells minimizing the impairment on surfactant production. There are various triggers for ARDS, and differences in the initial insult combined with underlying conditions may result in the activation of different inflammatory mechanisms. Based on the aforementioned, we hypothesized that sevoflurane and isoflurane may act differently depending on the etiology of ARDS.

## Objectives

To compare the effects of sevoflurane with isoflurane on lung mechanics and histology in experimental pulmonary (p) and extrapulmonary (exp) ARDS.

## Methods

Twenty-four Wistar rats (300-350 g) were randomly allocated to receive *Escherichia coli* lipopolysaccharide intratracheally (200 µg, ARDSp) or intraperitoneally (1,000 µg, ARDSexp). After 24 h, animals were randomized into subgroups anesthetized with sevoflurane (1 MAC) or isoflurane (1 MAC). All animals were paralyzed and protective mechanically ventilated with V_T_ = 6 ml/kg, RR = 80 bpm, PEEP = 3 cmH_2_O, and FiO_2_ = 0.4 for 1 h. Lung mechanics and arterial blood gases were analyzed at baseline and after 1 h anesthesia. At the end of the experiments, lungs were removed for histological and molecular biology analysis. A549 cells, alveolar type II epithelial cell line, were incubated with *E. coli* LPS, followed by treatment or not with sevoflurane and isoflurane.

## Results

In ARDSp, sevoflurane reduced lung static elastance compared to baseline and was associated with lower alveolar collapse. Isoflurane resulted in no mechanical and morphological changes. In ARDSexp, both sevoflurane and isoflurane did not alter lung mechanics. Arterial blood gases did not differ between the different anesthetic agents and ARDS groups. Sevoflurane presented lower expression of interleukin (IL)-6 and pro-caspase 3 and higher surfactant protein (SP)-B expression compared to isoflurane in ARDSp. In ARDSexp, IL-6, pro-caspase 3 and SP-B expressions did not differ between sevoflurane and isoflurane. Additionally, sevoflurane increased the expression of SP-B in E. coli LPS-injured A549 cells.

## Conclusions

Sevoflurane attenuates lung mechanics and atelectasis, probably through the stimulation of surfactant release in ARDSp, but no morphofunctional and biological parameters were affected in ARDSexp. Isoflurane presented no significant effects in both ARDS groups.

## Grant Acknowledgment

FAPERJ, CNPq, CAPES, MS-DECIT

## References

[1] Voigtsberger S, Lachmann RA, Leuter AC, Schlapfer M, Booy C, Reyes L (2009). Sevoflurane ameliorates gas exchange and attenuates lung damage in experimental lipopolysaccharide-induced lung injury. Anesthesiology.

[2] Yue T, Roth Z'graggen B, Blumenthal S, Neff SB, Reyes L, Booy C (2008). Postconditioning with a volatile anaesthetic in alveolar epithelial cells in vitro. Eur Respir J.

